# Cognitive Outcomes and Delirium After Cardiac Neurodevelopmental Program Implementation for Children With Congenital Heart Disease

**DOI:** 10.1001/jamanetworkopen.2024.56324

**Published:** 2025-01-24

**Authors:** Kelly R. Wolfe, Reagan Broach, Caelah Clark, Andrea Gerk, Sarah L. Kelly, Emily H. Maloney, Ariann Neutts, Hilary Patteson, Marisa Payan, Sarah Riessen, Sarah Watson, Sherrill D. Caprarola, Jesse A. Davidson

**Affiliations:** 1 Department of Pediatrics, University of Colorado Anschutz Medical Campus, Aurora; 2 Children’s Hospital Colorado, Aurora

## Abstract

**Importance:**

A recent advisory from the American Heart Association delineated the potential benefits of developmental care for hospitalized children with congenital heart disease (CHD) and a critical gap in research evaluating the association of such inpatient programs with neurodevelopmental outcomes.

**Objective:**

To investigate associations between the Cardiac Inpatient Neurodevelopmental Care Optimization (CINCO) program interventions, delirium, and neurodevelopment in young children (newborn through age 2 years) hospitalized with CHD.

**Design, Setting, and Participants:**

This cohort study used quality improvement data from inpatient cardiac units at a tertiary care children’s hospital in the US. Participants were children aged 0 to 2 years who were admitted for at least 7 days from September 1, 2018, to September 1, 2023. The CINCO program was implemented on September 1, 2020, in 6-month plan-do-study-act phases.

**Exposures:**

The 5 CINCO interventions were medical and/or nursing order panels, developmental kits, bedside developmental plans, caregiver mental health support handouts, and developmental care rounds.

**Main Outcomes and Measures:**

Number of days with delirium per patient, which was measured using the Cornell Assessment of Pediatric Delirium (a score higher than 9 indicated delirium). Neurodevelopment was measured using the Bayley Scales of Infant and Toddler Development, Fourth Edition (BSID-4).

**Results:**

The full sample included 1331 qualifying admissions for 1019 unique pediatric patients (median [range] age at admission, 3.65 [0-34.62] months; 771 males [57.9%]), with a subcohort of 121 unique patients (median [range] age at admission, 0.00 [0-9.85] months; 77 males [63.6%]) whose initial hospitalization occurred before age 10 months and who underwent BSID-4 evaluation at age 12 months or older. The mean (SD) number of days with delirium per patient was stable for 2 years prior to CINCO implementation, decreased by 54.0% between phases 1 and 2 of the CINCO program (from 3.05 [0.60] to 1.38 [0.21]), and then remained stable over time. Each of the 5 CINCO interventions was associated with lower delirium after false discovery rate (FDR) correction (eg, medical and/or nursing order panel: B = –1.376 [95% CI, –1.767 to –0.986]; *F*_2,1273_ = 47.767; partial η^2^ = 0.036; *P* < .001). Mean (SD) BSID-4 cognitive index scores were stable for 2 years prior to implementation, higher between phases 1 and 2 of the program (from 81.67 [14.14] to 93.92 [19.43]), and then remained stable over time. Four of the 5 interventions were associated with higher BSID-4 cognitive scores after FDR correction (eg, bedside developmental plans: B = 8.585 [95% CI, 2.247-14.923]; *F*_5, 101_ = 7.221; partial η^2^ = 0.067; *P* = .008). Delirium was associated with lower BSID-4 cognitive scores. There were no associations between delirium and BSID-4 language or motor scores.

**Conclusions and Relevance:**

This cohort study found that among hospitalized children with CHD, the implementation of an inpatient developmental care program was associated with reduced incidence of delirium and higher cognitive scores. Pediatric cardiac centers may consider adopting these low-cost, low-risk, generalizable program interventions.

## Key Points

**Question:**

Is developmental care for hospitalized children with congenital heart disease (CHD) associated with neurodevelopmental outcomes?

**Findings:**

In this cohort study of 1331 pediatric admissions for 1019 children with CHD at a single hospital, implementation of a systematic inpatient cardiac neurodevelopmental care program over 3 years was associated with reduced delirium and higher cognitive scores.

**Meaning:**

Findings of this study suggest that cardiac inpatient neurodevelopmental care practices may be associated with a lower incidence of delirium and better cognitive outcomes among children with CHD at age 12 to 39 months.

## Teaser

This cohort study examines the neurodevelopment and incidence of delirium among children aged 0 to 2 years with congenital heart disease (CHD) receiving 1 or more cardiac neurodevelopmental interventions during hospitalization.

## Introduction

Congenital heart disease (CHD) is the most common birth defect, occurring in approximately 1% of live births.^1^ Of these newborns, approximately 25% have critical CHD requiring surgical or catheterization intervention during infancy.^2^ Neurodevelopmental challenges are the most common long-term sequelae of critical CHD, with greater than 50% of children showing developmental delays in early childhood.^3^ Developmental delays often evolve into lifelong difficulties with attention and executive functioning, social challenges, and in some cases, reduced overall intellectual quotient.^4,5,6,7^ For adults with CHD, neurodevelopmental impairment is related to diminished quality of life, increased depression and anxiety symptoms, and lower socioeconomic status.^8,9^ Societal resource use is high for individuals with CHD due to neurodevelopmental impairment, with up to 65% receiving remedial academic or behavioral services and 50% using therapeutic services.^3,10^

While the factors in neurodevelopmental risks and resiliency for individuals with CHD are not fully understood, longer length of stay (LOS) during early interventional hospitalizations has been consistently associated with poorer neurodevelopmental outcomes.^11,12,13^ In efforts to address this factor, inpatient cardiac neurodevelopmental care programs have been increasingly implemented in pediatric cardiac centers.^14^ A recent science advisory from the American Heart Association described the potential conceptual benefits of inpatient developmentally focused cardiac care based on research in other settings (eg, the neonatal intensive care unit).^15^ The authors described a critical need for research elucidating the potential benefits of such programs in neurodevelopmental outcomes of infants and children with CHD.

In 2020, our center (Children's Hospital Colorado) initiated the Cardiac Inpatient Neurodevelopmental Care Optimization (CINCO) program. Initial research described the design of the CINCO program and the feasibility and sustainability of its implementation.^16^ The present study aimed to investigate the associations between CINCO interventions, delirium, and neurodevelopment in children (from newborn through age 2 years) hospitalized with CHD.

## Methods

This longitudinal cohort study was conducted at a tertiary care children’s hospital in the US. The CINCO program was approved by the Children's Hospital Colorado’s quality improvement review panel. The Colorado Multiple Institutional Review Board approved this study as a secondary use protocol to conduct research using quality improvement data and waived the informed consent requirement because of the retrospective nature of the study. We followed the Strengthening the Reporting of Observational Studies in Epidemiology (STROBE) reporting guideline.^17^

### Participants

Inclusion criteria were children aged 0 to 2 years who were admitted for at least 7 days to the cardiac inpatient units (including the cardiac intensive care unit [ICU] and the cardiac progressive care unit) from September 1, 2018, to September 1, 2023. For analyses involving neurodevelopmental outcomes, additional inclusion criteria were patients with an admission lasting at least 7 days prior to 10 months of age and who had undergone neurodevelopmental testing at 12 months of age or older. There were no exclusion criteria.

### Procedures

The CINCO program is a comprehensive, interdisciplinary cardiac inpatient neurodevelopmental care program consisting of 5 pillars of interventions: developmental kits, medical and/or nursing order panel (in the hospital’s electronic health record [EHR]), bedside developmental plans, caregiver mental health support handouts, and developmental care rounds. It was formally implemented on September 1, 2020, and previous work has described increasing implementation rates over multiple 6-month plan-do-study-act cycles.^16^ The CINCO program was created to be sustainable, generalizable, and low cost, with each of the 5 pillars interacting synergistically with one another to embed a neurodevelopmental approach within the culture and daily practices of the cardiac inpatient care teams.

### Data Collection 

All data were extracted automatically from the EHR and uploaded to Research Electronic Data Capture (REDCap).^18^ Data were collected at the level of each admission, including patient demographic and medical data. Sex assigned at birth was obtained from the EHR. Race and ethnicity data were also obtained from the EHR; this information is updated by the parent or guardian and was collected given the known implications of social determinants of health for neurodevelopmental outcomes in children with CHD.^19^ Scores from the Cornell Assessment of Pediatric Delirium (CAPD; score range: 0-32, with higher scores indicating more symptoms of delirium)^20^ are logged by bedside nurses into EHR flowsheets as routine clinical practice in cardiac inpatient units and were extracted automatically for each admission. The number of days with delirium per patient was calculated in 2 ways: a cutoff of CAPD score higher than 9 was selected as the primary outcome to maximize specificity^21^; a secondary analysis was completed using a cutoff of CAPD score higher than 8 to prioritize sensitivity.

Individual patient-level data included neurodevelopmental outcomes measured with the Bayley Scales of Infant and Toddler Development, Fourth Edition (BSID-4; score range: 45-155, with higher scores indicating better development),^22^ which was completed in the outpatient cardiac neurodevelopmental follow-up clinic, and entered into EHR flowsheets as part of routine clinical practice. The BSID-4 assesses early childhood development across 3 primary areas, yielding cognitive, language, and motor index standard scores.^22^

### Statistical Analysis

Data were examined for meeting statistical assumptions, including normality of distribution, homoscedasticity, multicollinearity of independent variables, and patterns of missing data. Missing data were dealt with using pairwise deletion. All admissions meeting the inclusion criteria were included in the analyses of CAPD scores, including patients with multiple qualifying admissions during the study period. For analyses with BSID-4 outcomes, we included only each patient’s first admission prior to 12 months of age, along with only their most recent BSID-4 evaluation completed after 12 months of age. This approach ensured that no duplicate patients were included and that the hospital admission preceded the BSID-4 evaluation.

Generalized linear models (analysis of variance) with follow-up simple contrasts were used to evaluate changes in CAPD and BSID-4 scores over time. Analysis of covariance was used to investigate associations between CINCO interventions, CAPD scores, and BSID-4 outcomes. For CAPD analyses, the sole a priori covariate was ICU LOS, a known risk factor for delirium.^23^ For BSID-4 analyses, 4 a priori covariates were included based on their known associations with neurodevelopmental outcomes: prematurity, defined as gestational age of less than 37 weeks at birth (yes or no)^24^; Society of Thoracic Surgeons-European Association for Cardio-Thoracic Surgery (STAT; score range: 1-5, with higher scores indicating greater risk of mortality associated with the surgical procedure) surgical score^25^; hospital LOS, in days^11,12,13^; and diagnosis (yes or no) of 1 of the 9 most common genetic syndromes associated with CHD (DiGeorge syndrome, Noonan syndrome, Trisomy 21, Williams syndrome, Leopard syndrome, Turner syndrome, Alagille syndrome, Kabuki syndrome, and Jacobsen syndrome).^26^ A Benjamini-Hochberg correction was used to correct for multiple comparisons (false discovery rate <.05). Pearson correlation was used for post hoc analyses exploring the associations between patient characteristics and delivery of CINCO interventions.

Two-sided *P* < .05 indicated statistical significance. Data analysis was performed using IBM SPSS Statistics, version 29 (IBM Corp).

## Results

The full sample included 1331 admissions (1019 unique patients; median [range] age at admission, 3.65 [0-34.62] months; 560 females [42.1%] and 771 males [57.9%]) over the 5-year study period (Table 1). Of these patients, 121 unique patients had BSID-4 outcome data after 12 months of age, with a first admission prior to 10 months of age (median [range] age at admission, 0.00 [0-9.85] months; 44 females [36.4%] and 77 males [63.6%]). The median (range) length of time between the index admission and BSID-4 evaluation was 19.3 (3.3-39.0) months. The number of CINCO interventions received by patients in the full cohort and BSID-4 subcohort is provided in the eTable in Supplement 1.

**Table 1.** Descriptive Data for Overall Sample and Subset With Neurodevelopmental Evaluations

Characteristic	Patients, No. (%)		Differences between groups, *P* value
	All qualifying admissions (n = 1331)	Unique patients with first hospitalization <10 mo and BSID-4 evaluation at >12 mo (n = 121)	
Sex assigned at birth			
Female	560 (42.1)	44 (36.4)	.10
Male	771 (57.9)	77 (63.6)	
Age at admission, median (range), mo	3.65 (0-34.62)	0.00 (0-9.85)	<.001
Race^a^			
American Indian or Alaska Native	42 (3.2)	2 (1.7)	.78
Asian	21 (1.6)	3 (2.5)	
Black	62 (4.7)	5 (4.1)	
White	851 (63.9)	81 (66.9)	
Other^b^	199 (15.0)	21 (17.4)	
Declined to answer	156 (11.7)	9 (7.4)	
Ethnicity			
Hispanic or Latinx ethnicity^a^	396 (29.8)	34 (28.1)	.91
No. of days with CAPD score >9 per patient, median (range)	0.00 (0-62.00)	1.00 (0-20.00)	.02
STAT surgical score			
5	128 (9.6)	46 (38.0)	<.001
4	322 (24.2)	41 (33.9)	
3	116 (8.7)	8 (6.6)	
2	301 (22.6)	22 (18.2)	
1	112 (8.4)	4 (3.3)	
Nonsurgical	352 (26.4)	NA	
Hospital LOS, median (range), d	9.00 (7.00-985.00)	42.37 (7.00-363.00)	<.001
ICU LOS, median (range), d	9.00 (0-281.00)	17.00 (1-120.00)	<.001
Genetic syndrome, yes	80 (3.5)	10 (8.3)	>.99
Premature birth, yes	438 (19.1)	17 (14.0)	.01
CINCO intervention^c^			
Medical and/or nursing order panel, yes	546 (64.8)	62 (80.0)	<.001
Developmental kits, yes	338 (41.0)	50 (67.0)	.002
Bedside developmental plans, yes	343 (41.8)	56 (74.6)	<.001
Developmental care rounds, yes	199 (24.0)	36 (48.0)	<.001
Caregiver mental health support handouts, yes	451 (54.0)	49 (65.0)	.12
Age at BSID-4 evaluation, median (range), mo	NA	19 (12-39)	NA
BSID-4			
Cognitive index standard score, mean (SD)	NA	87.32 (17.09)	NA
Language index standard score, mean (SD)	NA	85.02 (20.61)	NA
Motor index standard score, mean (SD)	NA	82.74 (15.00)	NA

Abbreviations: BSID-4, Bayley Scales of Infant and Toddler Development, Fourth Edition (score range: 45-155, with higher scores indicating better development); CAPD, Cornell Assessment of Pediatric Delirium (score range: 0-32, with higher scores indicating more symptoms of delirium); CINCO, Cardiac Inpatient Neurodevelopmental Care Optimization; ICU, intensive care unit; LOS, length of stay; NA, not applicable; STAT, Society of Thoracic Surgeons-European Association for Cardio-Thoracic Surgery (score range: 1-5, with higher scores indicating greater risk of mortality associated with the surgical procedure).

^a^ Race and ethnicity data were obtained from the electronic health record and were updated by the parent or guardian.

^b^ Other includes Native Hawaiian or Other Pacific Islander and multiracial.

^c^ CINCO intervention implementation rates were calculated only for patients whose hospitalization occurred during a CINCO implementation phase and excluded patients who were hospitalized during the pre-CINCO baseline period.

**Figure 1.**
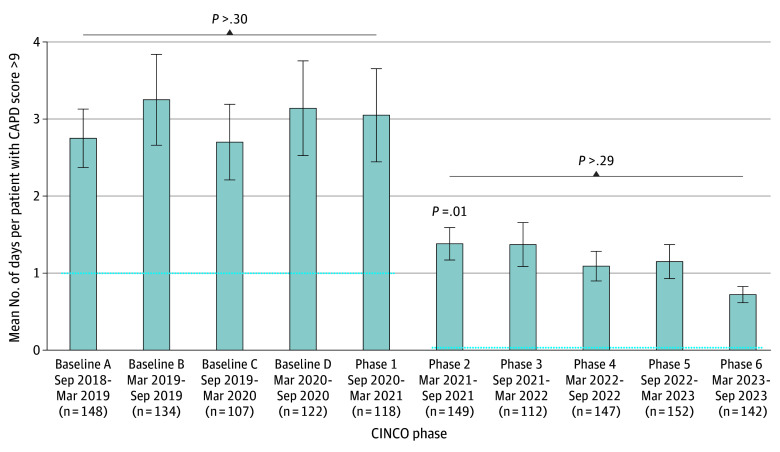
Days With Delirium Before Implementation and During the CINCO Program Baselines A through D were prior to the Cardiac Inpatient Neurodevelopmental Care Optimization (CINCO) program launch. Phases 1 to 6 represent the 6 plan-do-study-act quality improvement cycles of the CINCO program to date. Graph bars represent mean values, whiskers represent 95% CIs; and dotted lines represent the median value of days with delirium in each phase. CAPD indicates Cornell Assessment of Pediatric Delirium.

The mean number of days with a CAPD score higher than 9 per patient remained stable during the four 6-month baseline phases prior to CINCO implementation and during phase 1 of the CINCO program (Figure 1). There was a 54.0% reduction in delirium between phases 1 and 2 (3.05 [0.60] to 1.38 [0.21]; *P* = .01), which was sustained over time across phases 3 to 6. As CAPD scores were not normally distributed in the sample, median number of days with CAPD score higher than 9 was also examined. The median (range) number of days with delirium was 1 (0-62) for all baseline phases and phase 1 and was 0 (0-20) (*P* < .001) for phases 2 to 6. Follow-up analyses showed reduced delirium incidence associated with receiving each of the 5 CINCO interventions, although effect sizes were modest for each individual intervention (Table 2). For example, receiving the medical and/or nursing order panel was associated with reduced delirium with a small effect size (B = –1.376 [95% CI, –1.767 to –0.986]; *F*_2,1273_ = 47.767; partial η^2^ = 0.036; *P* < .001).

**Table 2.** Associations Between Receiving CINCO Interventions and Delirium Score

CINCO intervention^a^	CAPD score			*P* value^c^
	B (95% CI)	*F*_2,1273_	Partial η^2^^b^	
Medical and/or nursing order panel	−1.376 (−1.767 to −.986)	47.767	.036	<.001
Developmental kits	−1.195 (−1.640 to −.751)	27.825	.021	<.001
Bedside developmental plans	−1.267 (−1.710 to −.824)	31.479	.024	<.001
Developmental care rounds	−1.518 (−2.063 to −.974)	29.904	.023	<.001
Caregiver mental health support handouts	−1.050 (−1.455 to −.645)	25.862	.020	<.001

Abbreviations: CAPD, Cornell Assessment of Pediatric Delirium (score range: 0-32, with higher scores indicating more symptoms of delirium); CINCO, Cardiac Inpatient Neurodevelopmental Care Optimization.

^a^ The covariate in the general linear model was intensive care unit length of stay.

^b^ Partial η^2^ is a measure of effect size, with 0.01 indicating small effect size; 0.06, medium effect size; and 0.14, large effect size.

^c^ *P* value significant at false discovery rate <.05.

When these analyses were repeated using a CAPD score cutoff higher than 8, the results were similar. Specifically, the mean (SD) number of days with a CAPD score higher than 8 remained stable during the baseline phases and phase 1 (eg, baseline A and phase 1: 3.34 [5.16] and 3.92 [8.45] days; *P* > .52) and decreased between phases 1 and 2 (3.92 [8.45] and 1.75 [3.23] days; *P* = .01); this reduction was sustained across phases 3 to 6 (phase 3: 1.60 [3.28], phase 4: 2.65 [3.12], phase 5: 1.43 [3.25], phase 6: 0.85 [1.40] days; *P* > .12). Again, follow-up analyses showed that each CINCO intervention was independently associated with reduced delirium using the CAPD score cutoff higher than 8.

**Figure 2.**
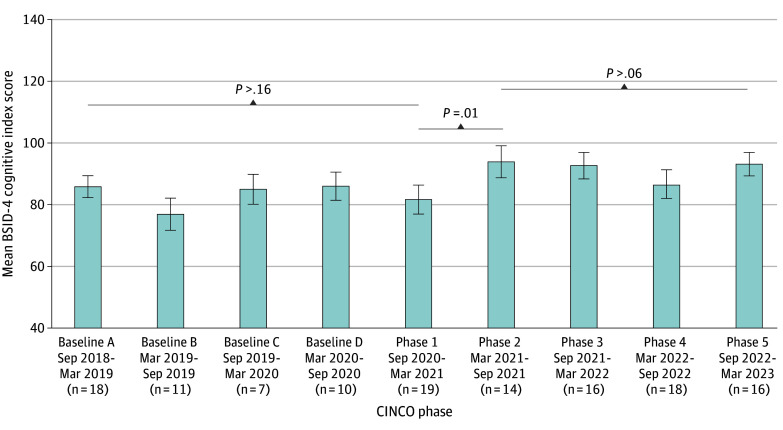
Cognitive Index Scores After 12 Months of Age by First Surgical Admission Occurring During the CINCO Program Graph bars represent mean values, and whiskers represent 95% CIs. BSID-4 indicates Bayley Scales of Infant and Toddler Development, Fourth Edition; CINCO, Cardiac Inpatient Neurodevelopmental Care Optimization.

BSID-4 cognitive index standard scores at 12 months of age or older increased over the course of the study period, grouped by the CINCO implementation phase in which the initial hospitalization occurred, controlling for the 4 a priori covariates (overall model, *F* = 2.53; *P* = .03) (Figure 2). Mean (SD) cognitive index scores remained stable during the 4 baseline phases approximately 1 to 1.5 SDs below the mean (from 85.83 [15.17] in baseline A to 86.00 [14.49] in baseline D) prior to CINCO implementation and in phase 1 (81.67 [14.14]) of the CINCO program and increased in phase 2 up to the clinically mean range (93.92 [19.43]; *P* = .01); this increase was sustained through phase 5 (93.12 [15.15]). Admissions during phase 6 were excluded because of the recency of this phase (ie, only 3 children whose first hospitalization occurred during phase 6 had returned for their BSID-4 evaluation after age 12 months, during the statistical analyses for this study). In a direct-comparison analysis of covariance grouping all index hospitalizations occurring prior to implementation and during the CINCO program, the mean (SD) BSID-4 cognitive index score was higher for the group whose hospitalization occurred during vs before implementation (90.57 [16.66] vs 81.86 [15.72]; *P* = .002). Univariate analyses examining BSID-4 language and motor index scores did not show significant change over time.

Four of the 5 CINCO interventions, when received during the patient’s first admission prior to 10 months of age, were independently associated with higher BSID-4 cognitive scores at 12 months of age or older, following a false discovery rate correction, with medium effect sizes (Table 3). For example, receiving bedside developmental plans was associated with higher cognitive scores with a medium effect size (B = 8.585 [95% CI, 2.247-14.923]; *F*_5, 101_ = 7.221; partial η^2^ = 0.067; *P* = .008) and receiving developmental kits was associated with higher cognitive scores with a large effect size (B = 10.535 [95% CI, 4.462-16.607; *F*_5, 101_ = 11.843; partial η^2^ = 0.105; *P* < .001). Post hoc partial correlations demonstrated an association between higher incidence of delirium (CAPD score >9) before 10 months of age and lower cognitive index score at 12 months of age or older (*r* = −0.259; *P* = .008), controlling for the 4 a priori covariates. This association was also observed when analyses were repeated using the cutoff of CAPD score higher than 8 (*r* = −0.363; *P* < .001).

**Table 3.** Associations Between Receiving CINCO Interventions During Initial Hospitalization Up to 9 Months of Age and Cognitive Index Score After 12 Months of Age

CINCO intervention^a^	BSID-4 cognitive index standard score			*P* value
	B (95% CI)	*F*_5,101_	Partial η^2^^b^	
Medical and/or nursing order panel	8.992 (2.487-15.497)	7.519	.069	.007^c^
Developmental kits	10.535 (4.462-16.607)	11.843	.105	<.001^c^
Bedside developmental plans	8.585 (2.247-14.923)	7.221	.067	.008^c^
Developmental care rounds	9.598 (2.382-16.814)	6.962	.064	.01
Caregiver mental health support handouts	8.696 (2.655-14.737)	8.154	.075	.005^c^

Abbreviations: BSID-4, Bayley Scales of Infant and Toddler Development, Fourth Edition (score range: 45-155, with higher scores indicating better development); CINCO, Cardiac Inpatient Neurodevelopmental Care Optimization.

^a^ Covariates in the general linear model (analysis of covariance) included Society of Thoracic Surgeons-European Association for Cardio-Thoracic Surgery surgical score, prematurity (yes or no), presence of a genetic syndrome (yes or no), and hospital length of stay.

^b^ Partial η^2^ is a measure of effect size, with 0.01 indicating small effect size; 0.06, medium effect size; and 0.14, large effect size.

^c^ *P* value significant at false discovery rate <.05.

Additional post hoc analyses evaluated associations between known sociodemographic and medical risk factors for worse neurodevelopmental outcomes and receiving CINCO interventions. Younger patients at the time of admission were more likely to receive medical and/or nursing order panel (*r* = −0.303; *P* < .001), bedside developmental plans (*r* = −0.212; *P* = .021), and developmental kits (*r* = −0.249; *P* = .006). Patients with a higher STAT surgical score were more likely to receive medical and/or nursing order panel (*r* = 0.237; *P* = .009) and developmental care rounds (*r* = 0.200; *P* = .03). Patients with a longer hospital LOS were more likely to receive medical and/or nursing order panel (*r* = 0.352; *P* < .001), bedside developmental plans (*r* = 0.321; *P* < .001), developmental kits (*r* = 0.253; *P* = .006), and developmental care rounds (*r* = 0.384; *P* < .001). There were no associations found between race and ethnicity, sex, presence of a genetic syndrome, or premature birth and receiving any CINCO intervention.

## Discussion

To our knowledge, this is the first study to show associations between cardiac inpatient neurodevelopmental care practices and improvements in clinical delirium scores as well as cognitive outcomes in children with CHD at age 12 to 39 months. These findings have fundamental implications for future clinical standards of care and avenues for research.

In the inpatient cardiac units, the mean (SD) number of days with delirium per patient was stable for 2 years prior to CINCO implementation, decreased by 54.0% (from 3.05 to 1.38) between phases 1 and 2, and was further reduced to less than 1 day by phase 6. Delirium is a common complication in the pediatric cardiac ICU, particularly in younger patients, and is associated with longer ICU stays.^23^ Preventive approaches for pediatric delirium include promotion of sleep-wake cycles, deintensification of care, and early mobilization,^27^ all of which are included in the CINCO program, which may help explain the associations between delirium and CINCO interventions. These associations are noteworthy given that children who received CINCO interventions were also more likely to have characteristics that placed them at higher risk for delirium, such as younger age, longer LOS, and higher cardiac acuity. If the analyses were confounded by patient-specific risk factors, the anticipated effect would have been in the opposite direction.

Treatment for pediatric delirium often involves potentially neurotoxic exposures with antipsychotic medications, while treatment for accompanying agitation may involve benzodiazepines and/or opioids; each of these may be harmful for the developing brain.^28,29^ Our findings support an association between the number of days with delirium during the initial hospitalization and cognitive outcomes at age 12 to 39 months, after adjusting for known risk factors for neurodevelopmental delays, including hospital LOS, premature birth, genetic syndrome, and STAT surgical score. Research on the neurodevelopmental impact of pediatric delirium is limited. One study conducted in the pediatric ICU found no relationship between severity of delirium and neuropsychological outcomes, although the authors noted that their findings may have been limited by their sample’s modest size (n = 47) and wide age range (1-16 years) as well as variability in presenting medical conditions.^30^ Novel findings of the present study suggest that nonpharmacologic approaches to delirium prevention and/or treatment, in the context of developmentally supportive inpatient care practices, may be beneficial for cognitive outcomes.

In addition to reductions in delirium, cognitive scores were higher over the course of CINCO implementation, adjusting for important risk factors. For children with CHD whose first interventional hospitalization occurred during the 2 years prior to CINCO implementation, mean BSID-4 cognitive index standard scores at 12 months or older measured 1 to 1.5 SDs below the means. This score improved to the clinical average range between phases 1 and 2. While some degree of inherent variability in neurodevelopmental scores is expected over time, particularly with younger age at testing,^31^ variations in mean BSID-4 cognitive index scores across phases 2 to 5 were not statistically significant, suggesting maintenance of overall gains. Historical data indicate that cognitive outcomes for young children with CHD have not meaningfully improved over time concomitantly with increases in overall survival.^32^ Follow-up analyses showed bivariate associations between 4 of the 5 CINCO interventions and cognitive outcomes at age 12 to 39 months after adjusting for known risk factors and correcting for multiple comparisons. If replicated in future studies, these findings would represent a paradigm shift in cardiac neurodevelopmental care, outcomes, and research, in which the need for effective interventions to improve neurodevelopmental trajectories has been emphasized but has not yet been addressed.^33^

### Limitations

This study has several limitations. A crucial consideration when interpreting the results is that the variables used to quantify CINCO implementation may represent only a small part of the broader culture change that has taken place at our institution to embrace neurodevelopmentally focused cardiac inpatient care, with the CINCO program serving as the impetus and sustaining force. For example, the caregiver mental health support handouts are provided in the context of a 1:1 social work consultation, during which emotional support is provided, resource needs are addressed, and referrals for direct mental health services are offered to caregivers as needed; the impact of this CINCO intervention likely extends beyond the written informational handouts. Bedside developmental plans and developmental kits may serve as a proxy for more frequent physical, occupational, or speech-language therapy during hospitalization, in addition to their unique role as therapy extenders.^16^ The CINCO program also includes a specialized cadre of volunteers who hold and play with hospitalized infants and toddlers with CHD and who perform other developmental plan activities. While the data suggest that CINCO volunteer shifts have increased over time, we were not able to assess the direct implications of volunteer shifts for patient-specific delirium or cognitive outcomes because the volunteers cannot document in the EHR.

Another limitation is the observational cohort design, which precludes the identification of causality among associations. Only about 12% of the hospitalized cohort returned for outpatient neurodevelopmental follow-up at age 12 to 39 months. This low percentage is due in part to the limited number of appointments available in this clinic, the center’s 7-state catchment area, and the CINCO program’s intentional prioritization of high-risk patients for follow-up appointments. As a result, there is uncertainty about the extent to which associations between CINCO interventions and higher cognitive scores may generalize to patients with mild CHD. Future research should examine whether focusing inpatient neurodevelopmental care resources on high-risk patients might achieve similar overall results. In addition, because many of the patients in this sample receive outpatient developmental therapies (eg, physical, occupational, and speech-language therapies) through state-based early intervention services or home health companies, which are not documented in the EHR, this information was not available for inclusion in our analyses. Furthermore, because the 5 CINCO interventions were interrelated (ie, if a patient received one intervention, there was a high chance they received others) and all started in the same time frame, we could not assess which aspect of the program may have had the greatest role in delirium or cognitive outcomes in the general linear models (ie, due to multicollinearity of independent variables). Our approach simultaneously targeted multiple variables inherent to hospitalization for children with CHD and associated with delirium and neurodevelopmental delays through a comprehensive, interdisciplinary program that included caregiver support and patient-focused interventions. We leveraged existing resources and team members and created interdependent processes that integrated into clinical workflows to aid implementation and generalizability but were not rigorously controlled as in a clinical trial.

## Conclusions

The initial findings of this study demonstrated associations between CINCO interventions, delirium scores, and cognitive outcomes among children with CHD aged 0 to 2 years. Specifically, the incidence of delirium was reduced and cognitive outcomes were improved after the implementation of the CINCO program. Pediatric cardiac centers may consider adopting these low-cost, low-risk, generalizable program interventions using existing personnel and resources. These results are promising and warrant replication studies at other centers. Future research should prioritize including additional measures of social determinants of health and methods to investigate causal pathways between inpatient neurodevelopmental care during infancy and improved delirium and later cognitive outcomes in children with CHD.

## Notes

Supplement 1. **eTable.** Number of CINCO Interventions Received During Hospitalization

Supplement 2.
Data Sharing Statement
