# Supplementary material for: Cognitive Outcomes and Delirium After Cardiac Neurodevelopmental Program Implementation for Children With Congenital Heart Disease
Source: JAMA Netw Open. 2025 Jan 24;8(1):e2456324. doi: 10.1001/jamanetworkopen.2024.56324 (PMC11762250; doi:10.1001/jamanetworkopen.2024.56324)
Supplement: Supplement 1. — eTable. Number of CINCO Interventions Received During Hospitalization [file jamanetwopen-e2456324-s001.pdf]

Supplemental Online Content

Wolfe KR, Broach R, Clark C, et al. Cognitive outcomes and delirium after cardiac neurodevelopmental program implementation for children with congenital heart disease. *JAMA Netw Open*. 2025;8(1):e2456324. doi:10.1001/jamanetworkopen.2024.56324

eTable. Number of CINCO Interventions Received During Hospitalization

This supplemental material has been provided by the authors to give readers additional information about their work.

© 2025 Wolfe KR et al. *JAMA Network Open.*

eTable. Number of CINCO Interventions Received During Hospitalization

CINCO Intervention	Overall Sample (N=1,331)	Subset with BSID-4 Follow-Up (N = 121)
Baseline (pre-CINCO)	511 (38.3%)	46 (38.0%)
0 CINCO Interventions	132 (9.9%)	2 (1.6%)
1 CINCO Intervention	142 (10.6%)	6 (4.9%)
2 CINCO Interventions	179 (13.4%)	7 (5.7%)
3 CINCO Interventions	181 (13.5%)	22 (18.1%)
4 CINCO Interventions	121 (9.0%)	25 (20.6%)
5 CINCO Interventions	65 (4.8%)	13 (10.7%)

© 2025 Wolfe KR et al. *JAMA Network Open.*
