# Supplementary material for: Cognitive Outcomes and Delirium After Cardiac Neurodevelopmental Program Implementation for Children With Congenital Heart Disease
Source: JAMA Netw Open. 2025 Jan 24;8(1):e2456324. doi: 10.1001/jamanetworkopen.2024.56324 (PMC11762250; doi:10.1001/jamanetworkopen.2024.56324)
Supplement: Supplement 2. — Data Sharing Statement [file jamanetwopen-e2456324-s002.pdf]

Data Sharing Statement

Wolfe. Cognitive Outcomes and Delirium After Cardiac Neurodevelopmental Program Implementation for Children With Congenital Heart Disease. *JAMA Netw Open*. Published January 24, 2025. doi:10.1001/jamanetworkopen.2024.56324

Data
Data available: No

Additional Information
Explanation for why data not available: Given that our data contains patient identifiers and is a retrospective dataset for which patient/family informed consent was waived, we are unable to share our hospitalization-level dataset.
